# Machine learning models diagnose oral squamous cell carcinoma based on cross-cohort oral microbial signatures

**DOI:** 10.3389/fmicb.2025.1692186

**Published:** 2026-01-06

**Authors:** Mingchao Wang, Yanfei Sun, Wen Gong

**Affiliations:** 1 Department of Preventive Dentistry, Qingdao Stomatological Hospital Affiliated to Qingdao University, Qingdao, China; 2 Center of Stomatology, Qingdao Municipal Hospital Group, Qingdao, China

**Keywords:** cross-cohort validation, machine learning, noninvasive diagnosis, oral microbiome, oral squamous cell carcinoma

## Abstract

**Introduction:**

The saliva microbiome of oral squamous cell carcinoma (OSCC) patients has been gradually unveiled, but there is a lack of cross-cohort studies, and there is no non-invasive diagnostic model across cohorts for OSCC.

**Methods:**

This study aimed to investigate the differences in saliva microbial composition between OSCC patients and healthy individuals using cross-cohort saliva microbiome data, comprising 354 healthy individuals and 311 OSCC patients (total *n*=665).

**Results:**

We found significant differences in saliva microbial composition between OSCC patients and healthy people. Seven microorganisms were significantly reduced and seven were significantly increased in OSCC patients, serving as potential biomarkers. Machine learning models, including Random Forest, Extra Trees, Gradient Boosting, and XGBoost, were constructed to diagnose OSCC using saliva microorganisms. These models achieved area under the curve (AUC) values ranging from 63.1% to 96.9% at both genus and species levels in a rigorous leave-one-cohort-out cross-validation.

**Discussion:**

Our study provides a robust non-invasive diagnostic model for OSCC and demonstrates that high diagnostic accuracy is achievable at both genus and species levels, suggesting that taxonomic resolution is not the primary limiting factor. Instead, the choice of model construction methods is crucial. Therefore, greater attention should be paid to the selection of model methods in clinical applications.

## 1 Introduction

Oral squamous cell carcinoma (OSCC) represents one of the most prevalent and aggressive forms of head and neck cancers, accounting for approximately 90% of all oral malignancies (Tan et al., 2023). With over 377,000 new cases and 177,000 deaths in 2020 (Hakim and Su, 2023), OSCC poses a significant clinical burden characterized by late-stage diagnosis and frequent recurrence (32.7%) (Zang et al., 2025). The disease’s complex etiology involves well-established risk factors, including tobacco use, alcohol consumption, and HPV infection (Smith et al., 2012; Santacroce et al., 2021).

Recent breakthroughs in microbiome research have revealed that the oral cavity harbors one of the most diverse microbial ecosystems in the human body, with distinct compositional shifts observed in OSCC patients (Gholizadeh et al., 2016; Kwak et al., 2024). Studies (Gonzalez Agurto et al., 2024; Su et al., 2024) demonstrate that salivary microbiota alterations may influence cancer progression through multiple mechanisms, including chronic inflammation induction, epithelial barrier disruption, and the production of carcinogenic metabolites. Particularly, species such as *Porphyromonas gingivalis* (Zang et al., 2025; Ibanez et al., 2020), *Fusobacterium nucleatum* (Sun et al., 2023; Munoz-Grez et al., 2025), and *Candida albicans* (Vadovics et al., 2021) have been implicated in OSCC development.

Current diagnostic approaches for OSCC, primarily visual examination and tissue biopsy, are not only invasive but also exhibit limited sensitivity in detecting early-stage lesions, highlighting the critical need for minimally invasive diagnostic alternatives (Pierfelice et al., 2024). However, while emerging research has explored salivary biomarkers as a potential solution (Wolf et al., 2017; Yang et al., 2018), these studies remain constrained by significant methodological limitations, including restricted sample sizes and an inadequate representation of diverse demographic populations. Therefore, cross-cohort integrated biomarker discovery and subsequent disease model construction are of paramount importance.

In this context, our study aims to bridge this gap by providing a comprehensive analysis of the saliva microbiome in OSCC patients and developing a robust, non-invasive diagnostic model. By leveraging advanced bioinformatics techniques and machine learning algorithms, we have identified a panel of microbial biomarkers that are significantly associated with oral cancer. Importantly, our findings demonstrate that the identification level of these biomarkers does not significantly impact the accuracy of the diagnostic model, suggesting that the choice of model construction method is a crucial factor in developing an effective diagnostic tool.

## 2 Methods

### 2.1 Study design and overview

**Figure 1:**
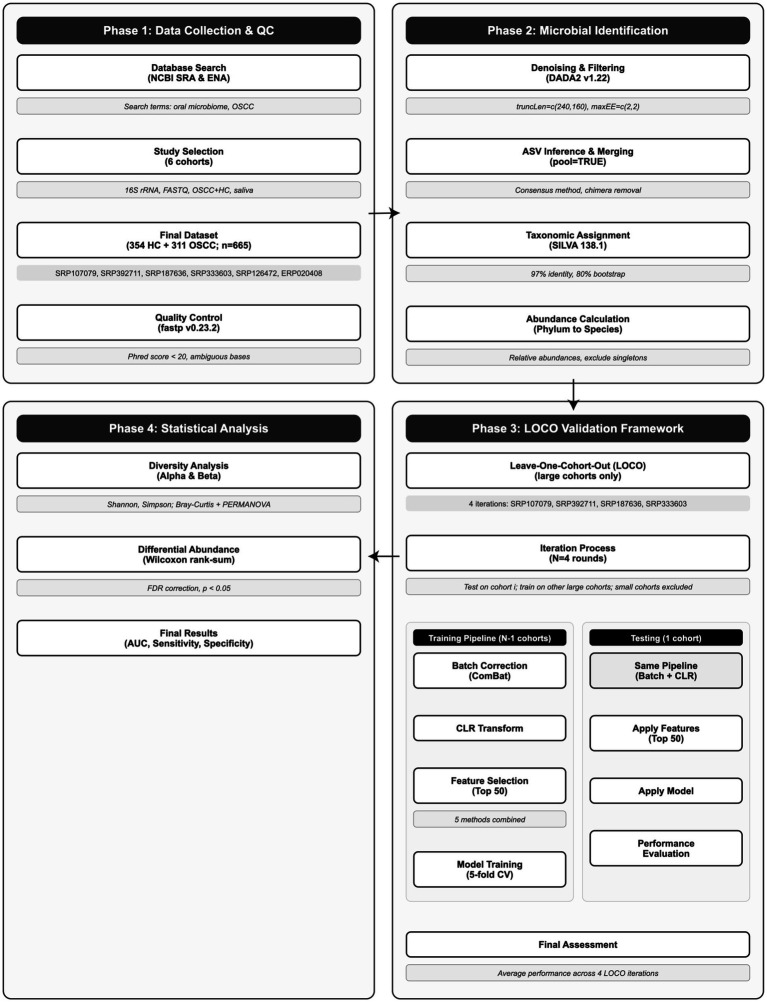
Overview of cross-cohort microbial classification with LOCO evaluation. Six saliva 16S cohorts (*n* = 665). Training applies ComBat batch correction, CLR transformation, integrated feature selection (top 50), and stratified 5-fold cross-validation; models are evaluated in the held-out cohort and summarized as the LOCO mean.

The comprehensive workflow of this study is illustrated in Figure 1. The process encompasses four main phases: data collection from public repositories, microbial identification, model training using a leave-one-cohort-out (LOCO) framework, and statistical analysis. This systematic approach ensures rigorous validation of microbial signatures across independent cohorts.

### 2.2 Data collection and quality check

We conducted a systematic search of public databases, including the National Center for Biotechnology Information (NCBI) Sequence Read Archive (SRA) and the European Nucleotide Archive (ENA), up to December 2023. Search terms included “oral microbiome,” “saliva microbiome,” “oral squamous cell carcinoma,” and “OSCC.” Our inclusion criteria were: (1) studies using 16S rRNA gene sequencing, (2) availability of raw sequence data (FASTQ format), (3) inclusion of both OSCC patients and healthy control groups, and (4) samples derived from saliva. Studies were excluded if they focused on other sample types (e.g., tissue biopsies and oral swabs), lacked a healthy control group, or did not provide accessible raw data. This screening process identified an initial eligible study. Studies utilizing anatomically matched normal sites from cancer patients (e.g., SRP097643) were excluded to strictly maintain healthy individuals as the control group. Ultimately, six independent cohorts meeting the inclusion criteria were selected for this study [NCBI SRA: SRP107079 (Lee et al., 2017), SRP392711, SRP187636 (Takahashi et al., 2019), SRP333603, SRP126472 (Vesty et al., 2018); ENA: ERP020408 (Wolf et al., 2017)]. This compiled dataset consisted of saliva microbiome data from 354 healthy individuals and 311 OSCC patients (Supplementary Table S1), providing a substantial and diverse sample size for identifying common microbial signatures. It is important to note that detailed metadata for potential confounding factors, such as tobacco use, alcohol consumption, medications, and comorbidities, were not uniformly available across all included cohorts, a common limitation in meta-analyses of public data. Reads with an average Phred score < 20 or containing ambiguous bases (N) were discarded using fastp v0.23.2 (Chen et al., 2018).

### 2.3 Microbial identification

DADA2 v1.22 was subsequently applied for further denoising (Callahan et al., 2016). Specifically, raw reads were filtered and trimmed using the filterAndTrim function with the following parameters: truncLen = c(240, 160), maxN = 0, maxEE = c(2, 2), truncQ = 2, and rm.phix = TRUE. After trimming, error rates were learned using the learnErrors function, and amplicon sequence variants (ASVs) were inferred using the dada function with the pool = TRUE option. Paired-end reads were subsequently merged. Chimeric sequences were identified and removed using the removeBimeraDenovo function with method = “consensus.” The resulting ASVs were imported into Parallel-META 3 (Jing et al., 2017) and aligned against the SILVA 138.1 database (Quast et al., 2013) using the default 97% identity threshold. Taxonomic ranks from kingdom to species were assigned with an 80% bootstrap confidence cutoff. Singleton ASVs and those unclassified at the phylum level were excluded. After profiling both taxonomy and function, we calculated sequence counts and relative abundances (ranging from 0 to 100%) for all OTUs and annotated taxa, spanning from the phylum to species levels.

### 2.4 Data preprocessing and feature selection

To rigorously evaluate model generalizability across different data sources, we implemented a leave-one-cohort-out (LOCO) cross-validation strategy. In this approach, each of the four cohorts was iteratively held out as an independent test set, while the remaining four cohorts were combined and used for training the models. This process was repeated five times, ensuring that each cohort was used for testing exactly once. This method provides a robust estimate of how the models will perform on new, unseen data from a different cohort. The training data, processed at both the genus and species levels, underwent a comprehensive preprocessing pipeline. To address potential batch effects arising from the multi-cohort design, we applied a ComBat-style algorithm to the training data in each fold of the cross-validation. This correction was essential to minimize non-biological, study-specific variation that could otherwise be confounded with true biological signals of OSCC. By standardizing the feature distributions across different cohorts, this step enhances the reliability and generalizability of the cross-cohort analysis, ensuring the model learns robust disease-associated patterns.

For feature selection, we developed an integrated ranking system to identify the most informative microbial signatures. To properly handle the compositional nature of the abundance data, we first applied a centered log-ratio (CLR) transformation. Features were scored using a weighted combination of five distinct metrics: (1) non-parametric differential abundance between OSCC and healthy controls using the Mann–Whitney U-test with FDR correction, (2) mutual information scores calculated via mutual_info_classif to capture non-linear relationships with the disease state, (3) feature importance derived from a Random Forest model (100 estimators), (4) feature importance from an XGBoost model, and (5) discriminative ability assessed by the ANOVA F-test with FDR correction. Each metric’s scores were standardized to a common scale, and a final composite score was calculated for each feature. The top 50 features with the highest composite scores were selected for subsequent model training.

### 2.5 Random Forest model

Random Forest Model A Random Forest classifier was implemented using scikit-learn with 261 trees (n_estimators = 261), maximum depth of 11, minimum sample split of 8, minimum sample leaf of 1, max_features set to “log2,” and bootstrap enabled.

### 2.6 Extra Trees model

An Extra Trees classifier was optimized using Optuna for hyperparameter tuning. The final parameters were configured as follows: number of estimators (n_estimators) ranges from 100 to 300, maximum depth (max_depth) between 3 and 12, minimum sample split (min_samples_split) between 2 and 20, minimum sample leaf (min_samples_leaf) between 1 and 10, and max_features selected from “sqrt” or “log2.”

### 2.7 Gradient Boosting model

Gradient Boosting Model A Gradient Boosting classifier was constructed using scikit-learn’s GradientBoostingClassifier with 882 estimators, a maximum depth of 13, a learning rate of 0.041, a subsample of 0.826, a minimum sample split of 12, and a minimum sample leaf of 1.

### 2.8 XGBoost model

XGBoost Model Extreme Gradient Boosting (XGBoost) was executed via the XGBoost Python package with 805 boosting rounds, a max_depth of 12, a learning_rate of 0.153, a subsample of 0.895, a colsample_bytree of 0.636, a reg_alpha of 3.773, and a reg_lambda of 3.812.

### 2.9 Statistical analyses

Statistical analyses were conducted entirely in R v4.3.1. Alpha-diversity (Shannon and Simpson) was calculated in the phyloseq package v1.44.0. Beta-diversity was assessed with Bray–Curtis using the ade4 package’s function. Group differences in beta-diversity were tested with permutational multivariate analysis of variance (PERMANOVA) using adonis2 (vegan v2.6-4, 9,999 permutations). Differentially abundant genera and species between groups were identified by two-sided Wilcoxon rank-sum tests with Benjamini–Hochberg false-discovery rate (FDR) correction. To account for the compositional nature of microbiome data, feature counts were first transformed using a centered log-ratio (CLR) transformation prior to statistical testing. Taxonomic features with an adjusted *p*-value of < 0.05 were considered statistically significant.

## 3 Results

### 3.1 Microbial composition on cross-cohort OSCC

**Figure 2:**
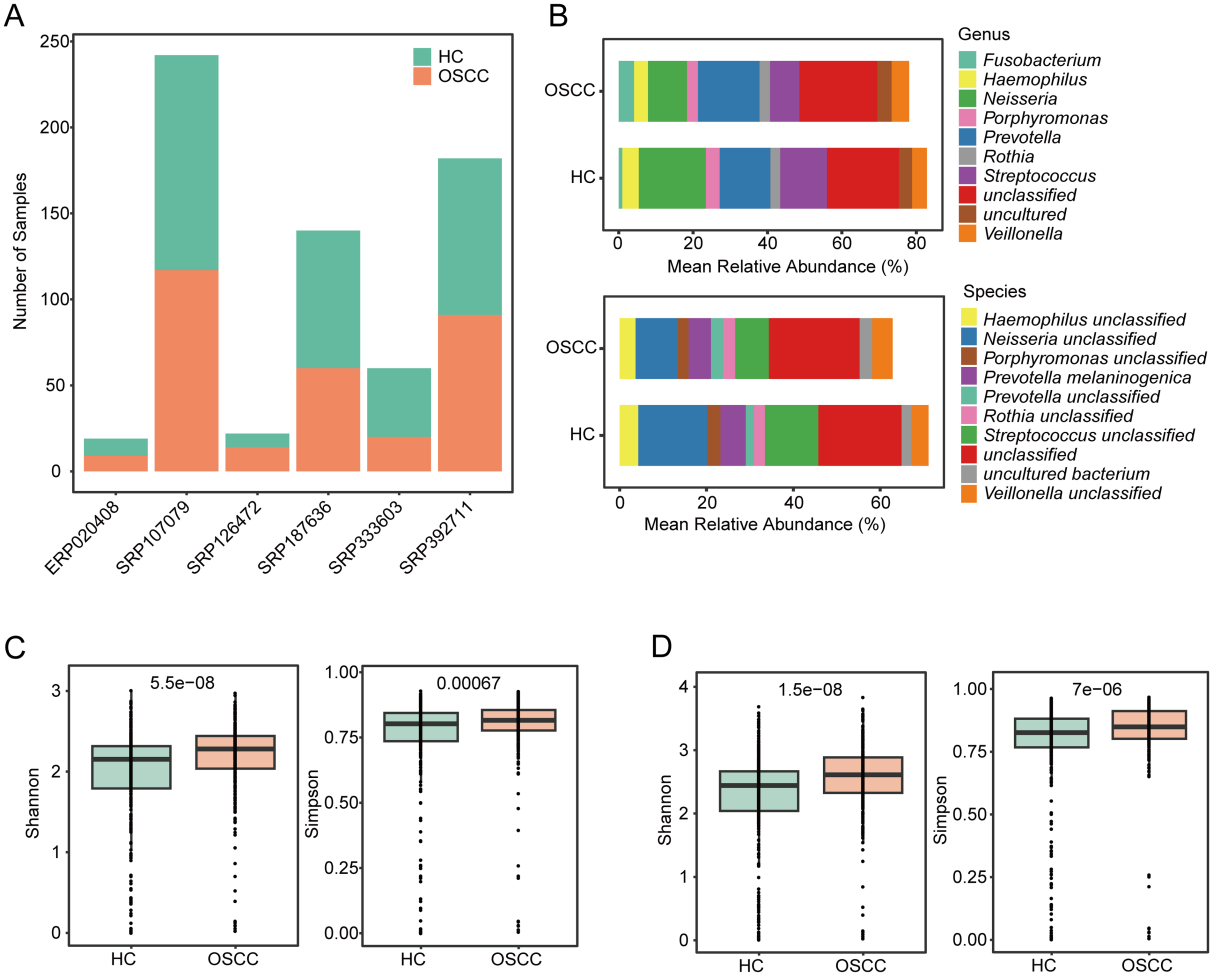
Salivary microbiome composition and diversity of oral cancer across cohorts. **(A)** Datasets included in this study. **(B)** Relative abundances of the top 10 bacterial genera and species across all samples. Shannon and Simpson diversity indices; significance was assessed by Wilcoxon rank-sum tests at the genus **(C)** and species level **(D)**.

To comprehensively characterize the oral microbiome composition in OSCC patients, we integrated 6 publicly available datasets comprising a total of 665 samples, including 354 healthy controls and 311 OSCC patients (Figure 2A; Supplementary Table S1). Subsequent genus and species-level analyses of microbial mean relative abundance across the three health states revealed dominant species (Figure 2B; Supplementary Tables S2, S3). Specifically, the most abundant genus includes *Neisseria*, *Prevotella*, *Streptococcus*, *Veillonella*, *Haemophilus*, *Rothia*, *Fusobacterium,* and *Porphyromonas*. Despite the imprecision in species-level annotation, it was inferred from the genus mentioned above. The results depicted in Figures 2C,D show a comparative analysis of the HC and OSCC groups using alpha diversity. We observed significant differences in saliva microbiome alpha of HC in patients with OSCC, both at genus (Figure 2C, Shannon, *p* = 5.5e-8; Simpson, *p* = 0.00067) and species levels (Figure 2D, Shannon, *p* = 1.5e-8; Simpson, *p* = 7e-6).

### 3.2 Significant differences in saliva microbial composition between oral cancer patients and healthy people

**Figure 3:**
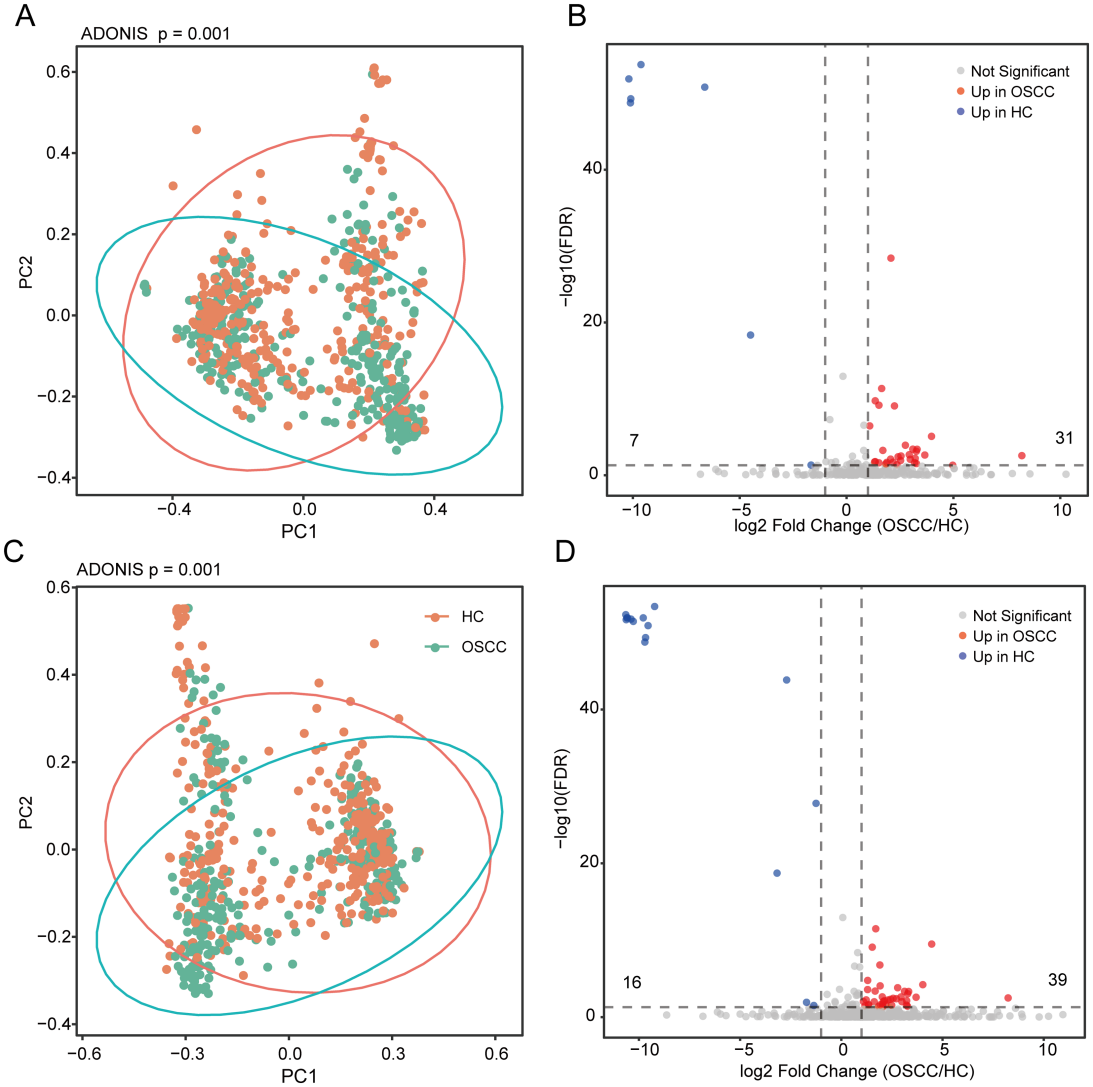
Beta diversity and differentially abundant taxa between oral cancer patients and healthy controls. **(A,C)** Beta-diversity comparison; PERMANOVA (adonis) was used to evaluate group differences. **(B,D)** Differences were tested by the Wilcoxon rank-sum test; *p*-values were FDR-adjusted, and an adjusted *p* < 0.05 and foldchange > 1 was considered statistically significant. **(A,B)** Represents the genus level, while **(B,D)** represents the species level.

Next, we focused on beta diversity and found significant differences in the saliva microbiome between HC and OSCC patients. The statistical significance of these separations is emphasized by an ANOVA test, indicating a highly significant difference (*p* < 0.001) in diversity measures between the HC and OSCC groups at the genus (Figure 3A) and species level (Figure 3C).

We further identified differentially abundant microbiota. At the genus level, 38 microbial taxa were significantly altered, with 31 being enriched and 7 depleted (e.g., *Fusobacterium* and *Parvimonas*) in OSCC (Figure 3B; Supplementary Table S4). At the species level, 55 were significantly altered, comprising 39 enriched and 16 depleted taxa (Figure 3D; Supplementary Table S5). All these microbial features represent potential biomarkers for OSCC.

### 3.3 Leave-one-cohort-out cross-validation reveals robust model performance

To assess the generalizability of our diagnostic models, we used a rigorous leave-one-cohort-out (LOCO) cross-validation scheme. Given that small-sample cohorts (*n* < 30, i.e., SRP126472 and ERP020408) may introduce high variance and lack representativeness as independent test sets, the LOCO validation was performed specifically on the four major cohorts (SRP107079, SRP392711, SRP187636, and SRP333603), while all data were utilized during the training phases.

**Figure 4:**
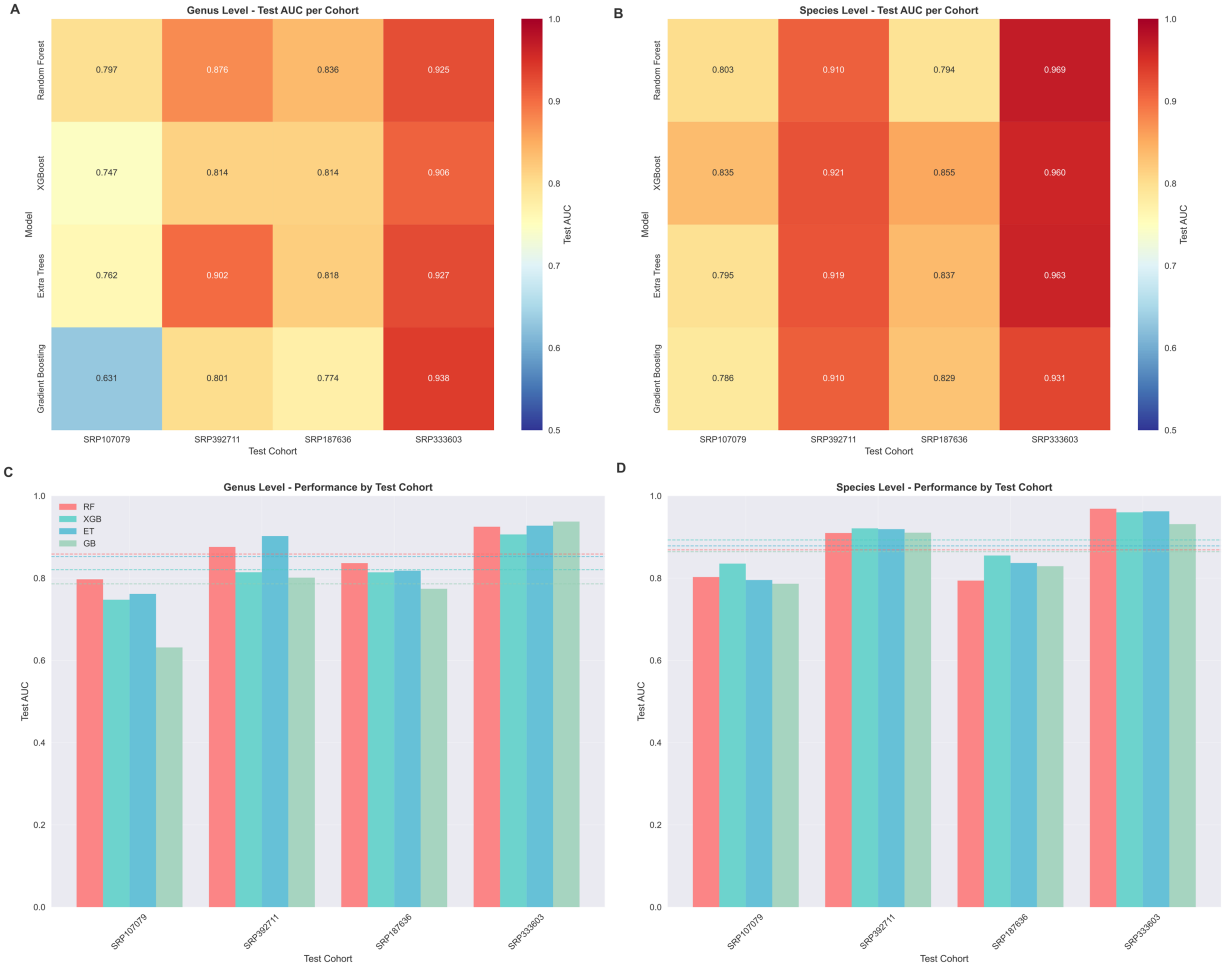
Comprehensive LOCO validation results: cohort-wise heatmap and performance comparison. **(A)** Genus-level test AUC across cohorts. **(B)** Species-level test AUC across cohorts. **(C)** Genus-level model performance by test cohort. **(D)** Species-level model performance by test cohort.

The performance of the four machine learning models was evaluated across the four cohorts, with results summarized as heatmaps of the Area Under the Curve (AUC) for testing sets. At the genus level, the models demonstrated strong generalization capabilities, though performance varied depending on the held-out cohort (Figures 4A,C). Notably, the Extra Trees model exhibited superior performance, achieving an AUC of 92.8% on the SRP333603 cohort and 90.2% on the large-scale SRP392711 cohort. Similarly, XGBoost showed robust results, with AUCs ranging from 81.4% (SRP187636) to 90.6% (SRP333603).

A similar robust pattern was observed at the species level (Figures 4B,D), where the models often achieved slightly higher accuracy. The Extra Trees model again performed exceptionally well, reaching an AUC of 96.3% in the SRP333603 cohort and 91.9% in SRP392711. XGBoost also demonstrated high stability, with AUCs consistently exceeding 83.0% across all major test cohorts (e.g., 92.1% for SRP392711 and 96.0% for SRP333603). Collectively, these results confirm that our study provides a robust non-invasive diagnostic model for OSCC. Importantly, high diagnostic accuracy was consistently achieved at both the genus (AUC up to 93.8%) and species (AUC up to 96.9%) levels. This demonstrates that with robust model construction, particularly when using ensemble methods such as Extra Trees, the predictive performance is stable and effective, independent of the specific taxonomic resolution.

## 4 Discussion

The oral microbiome is increasingly recognized as a key contributor to OSCC. with specific microbes and dysbiotic communities promoting tumorigenesis and metastasis through multiple mechanisms (Li et al., 2023; Li et al., 2022). The development of non-invasive diagnostic approaches for OSCC represents a crucial advancement in oncology (Pierfelice et al., 2024). The potential of oral microbiota as a diagnostic tool for oral cancer has gained increasing attention in recent years. For instance, Lee et al. (2017) identified a significant enrichment of *Parvimonas* and *Peptostreptococcus* in OSCC patients compared to healthy controls, suggesting their potential as microbial biomarkers. Similarly, Heng et al. (2022) demonstrated that a combined panel of 10 species could distinguish OSCC from a healthy control with high accuracy (86%). Yang et al. utilized 16S rRNA sequencing to reveal distinct microbial community shifts in saliva samples from OSCC patients, supporting the feasibility of microbiome-based screening, achieving 95.65% diagnostic accuracy (Yang et al., 2018). However, cross-cohort integrated biomarker discovery and subsequent disease model construction remain gaps. Most prior studies relied on single cohorts with limited sample sizes, which hindered the identification of universally applicable diagnostic signatures. In contrast, our study integrated 665 samples from 6 independent cohorts. This large-scale, multi-center dataset significantly enhances statistical power and allows for the identification of robust microbial signatures that are consistent across different populations and methodologies.

In our study, we developed a robust non-invasive diagnostic model for OSCC that holds significant promise for improving disease detection based on cross-cohort data. By implementing a rigorous leave-one-cohort-out cross-validation, we demonstrated that the models could generalize to unseen data, a critical step in clinical applicability. A critical aspect of our findings is the demonstration that the identification level of microorganisms, often considered potential biomarkers, does not have a substantial impact on the accuracy of the diagnostic model. This suggests that the intricacies of microbial taxonomy may not be as pivotal as previously thought in the context of oral cancer diagnosis. Instead, our results highlight the importance of the model construction method in developing an accurate and reliable diagnostic tool. Our comprehensive validation revealed that tree-based ensemble models, particularly Random Forest, XGBoost, and Extra Trees, consistently outperformed other tested algorithms. The superior performance of these ensemble methods suggests that the relationship between the oral microbiome and OSCC is characterized by complex, non-linear interactions—interactions that advanced tree-based architectures are uniquely suited to capture. By leveraging ensemble strategies—whether through bagging (as in Random Forest and Extra Trees) or boosting (as in XGBoost)—these models effectively balance bias and variance to mitigate overfitting. This robustness is particularly advantageous for handling high-dimensional and heterogeneous cross-cohort microbiome data.

The implications of this finding are profound, as it shifts the research focus from identifying specific biomarkers to optimizing model construction methods. This approach not only simplifies the diagnostic process but also enhances its accuracy and reliability, providing a new direction for future research in the field of oral cancer diagnosis.

However, translating this model into a clinical diagnostic tool requires addressing several practical challenges and acknowledging its current limitations. A key observation from our LOCO cross-validation is the performance heterogeneity across different test cohorts. For instance, while the models achieved excellent performance on the SRP333603 and SRP392711 cohorts (AUC > 0.9), the performance on SRP107079 was relatively lower (AUC ranged from 0.63 to 0.84). This variability likely stems from inter-cohort differences that persist despite our analytical approach, such as variations in population genetics, dietary habits, lifestyle factors (e.g., smoking and alcohol use), or technical artifacts from sample processing and sequencing protocols that create distinct batch effects.

Therefore, while our results are promising, several future steps are essential for clinical adoption. First, prospective validation in large, multi-center clinical trials is necessary to confirm the model’s real-world efficacy. Second, developing a standardized protocol for sample collection, processing, and sequencing is crucial for reproducibility and minimizing the batch effects observed in our study. We also acknowledge that potential confounding factors, such as tobacco and alcohol use, were not uniformly documented across the public datasets used. While this is an inherent limitation of meta-analyses, the robust performance of our model suggests that the identified microbial signatures are strong indicators of OSCC. To address these limitations and enhance generalizability, future studies must prioritize the systematic collection of such clinical metadata and include more diverse geographical regions and ethnic populations. This will be essential for building a universally applicable diagnostic tool and understanding the impact of these confounders. Finally, the cost-effectiveness and the integration of this bioinformatics pipeline into existing clinical workflows also represent key hurdles that need to be overcome.

Overall, our study identified a predictive microbial signature for OSCC across cohorts and constructed a non-invasive diagnostic model using saliva microbial composition. This model, through a rigorous leave-one-cohort-out validation, achieved test AUCs as high as 96.9%, underscoring that the choice of model construction method is a critical determinant of diagnostic performance. By highlighting the challenges of cohort heterogeneity and the necessity for methodological rigor, our study provides a robust foundation and a clear roadmap for the future development of microbiome-based diagnostics for oral cancer.

## Data Availability

The data presented in the study are deposited in the National Center for Biotechnology Information (NCBI) Sequence Read Archive (SRA) repository, accession numbers SRP107079, SRP392711, SRP126472, ERP020408, SRP333603, and SRP187636.
